# ParticipACTION: the future challenges for physical activity promotion in Canada

**DOI:** 10.1186/1479-5868-6-89

**Published:** 2009-12-09

**Authors:** Adrian Bauman, Nick Cavill, Lawrence Brawley

**Affiliations:** 1 Centre for Physical Activity and Health, School of Public Health, University of Sydney, Sydney, Australia; 2 Cavill Associates, Stockport, Cheshire, UK; 3 College of Kinesiology, University of Saskatchewan, Saskatoon, SK, Canada

## Abstract

This commentary is the concluding piece of a series of papers about the Canadian ParticipACTION initiative. It describes the resurgence of the new ParticipACTION as a national communications initiative in Canada, and sets this in an international context. The set of ParticipACTION papers in this issue establish benchmarks and provide baseline and initial impact data for the evaluation and monitoring of ParticipACTION, using qualitative and quantiative research methods. As a set, they describe a comprehensive approach to setting up evaluations of national social marketing efforts to promote physical activity.

## Commentary

The resurgence of the 'new ParticipACTION' in September 2007 was an important moment for physical activity promotion in Canada. The original ParticipACTION had lasted thirty years, and had become symbolic of quintessential Canadian values and beliefs about the benefits of participating in physical activity in the great outdoors. Political pressures and government cutbacks led to the withdrawal of funding in 2001, but six years later, it was re-born, with a short term mandate to develop communications strategies to promote physical activity, and develop partnerships and collaborations among key stakeholders involved in this area.

The purpose of this paper is to summarize and review the research presented in this series of papers in the light of ParticipACTION's current challenges. Further, we set this initiative in an international comparative context, indicating the unique opportunities it provides and the challenges that it faces in the future.

Health-related mass media campaigns to promote physical activity at the whole population level have been reported since the 1980s [1]. These have mostly been short-term persuasive campaigns, based on a communications-behavior change framework, and focused on influencing individual awareness, perceptions and physical activity behavior [2,3]. More recently, many of these have been described as 'social marketing', but few reflect a comprehensive set of marketing principles [4]. International policy frameworks have described the inter-sectoral nature of community-wide physical activity programs [5], the need to move beyond a health-centric approach, and the usefulness of engagement with municipal government, urban planning, Education, Sport, transport, and other sectors in planning initiatives [6]. The original ParticipACTION reflected many of these inter-sectoral principles, as well as being 'best practice' in social marketing efforts. It developed a branded and identified product, worked to understand what motivates consumers, and provided programs and services through a range of agencies and partnerships that met consumer preferences [7]. In addition, this 30 year public health campaign met the criteria for "good practice in mass media campaigns" [8], in that there was sustained communication, supported by public-private partnerships and by programs reaching geographically and demographically diverse population groups.

It is difficult to prove causal linkages, but the original ParticipACTION fostered the development of healthy environments for activity, and created a social norm around Canadians being an active population. In part, this was a counter reaction to the provocative 1970s ParticipACTION media message said that "the average 30 year old Canadian man is in about the same shape as the average 60 year old Swedish man" - [Participation Archive, [9]]. This message, although based largely on anecdote rather than science, has been credited with catalyzing the active living movement, fostering a Canadian sense of national pride, and contributed to ParticipACTION setting new social norms around outdoor activity. It is likely that this stimulus was a contributor to subsequent increases in physical activity in Canada seen since the 1980s, and rarely observed in other developed countries over such a sustained period [10].

The 'new ParticipACTION' was only launched in 2007, with modest governmental funds, a requirement to pursue large amounts of private sector funding, and a short tenure. The challenge for this re-incarnation is to engage with stakeholders, re-invent new population strategies, while keeping in mind the array of complex issues around physical inactivity, as well as promoting sport and recreation participation. The methods for achieving this may be partly mass communications, partly social marketing, and partly organizing and fostering partnerships across Canada to bring groups interested in population-level physical activity together. However, the complicating phenomenon, 'nature abhors a vacuum', led to much physical activity effort during the six-year hiatus during which ParticipACTION was quiescent. Many NGOs, Provincial Government strategies, the Coalition for Active Living and national level frameworks were developed, and received resources and attention. These ranged from the national Public Health Agency for Canada (PHAC) and the "Healthy living strategy" through to new Province-level campaigns (e.g., Healthy U, Alberta; Act Now BC in British Columbia; Saskatchewan's series of 'in Motion' programs, Active 2010 Ontario; Nova Scotia's Active Kids, Healthy Kids). This patchwork evolution of physical activity campaigns, and their lack of overall coordination, is one of the major challenges for the new ParticipACTION.

The series of research papers in this series of papers illustrate these challenges. Stakeholders need to be better engaged with the new ParticipACTION, and develop shared work programs and resource usage. Ideally, they would co-brand their physical activity work with the new ParticipACTION. The need for stakeholder agencies and professionals to engage is highlighted in the papers by Faulkner and by Plotnikoff [11,12]. One example of this is that although 96% of a selected sample of relevant stakeholders recognised the original ParticipACTION, only 55% of them identified the new one [12]. Further, some stakeholders reported potential overlap between their work and the new ParticipACTION, and reported low private sector engagement in physical activity programs [12]. Additional stakeholder research identified lack of leadership, lack of sustained funding, and a focus on obesity by governments and NGOs as critical issues [11] that inhibited stakeholders' promotion of physical activity. These findings, and the increasingly complex national and provincial policy environment, pose challenges for new efforts to coordinate physical activity efforts in Canada, and set baselines against which the new ParticipACTION's progress can be monitored. More effective physical activity outcomes are likely if a clear brand is disseminated and recognized nationally, if inter-sectoral partnerships share the advocacy and program development agenda, and if scarce resources are pooled to enhance program reach and scope [13].

The research in this issue also examined community awareness of ParticipACTION. This is an important initial outcome of mass communications [2], and effect sizes are usually much larger than for later, more distal campaign outcomes such as (physical activity) behavior change [14]. The study reported by Craig et al [15] showed that 57% of the population was aware of the new ParticipACTION's 2007 campaign targeting parents of young children. This evaluation also demonstrated higher ParticipACTION recall among women, more educated respondents, and those with active children, reflecting differential uptake patterns of many such campaigns [15]. The prompted recall rate following this specific campaign was higher than rates shown following large-scale physical activity campaigns in the UK (38%) or Australia (50%) [16,17], but lower than the 72% recall following the Verb campaign in the USA [18]. However, the scope of these campaigns differed widely in terms of both dissemination and funding also potentially accounting for some of the differences in recall rates.

These findings can be set in an international context, for example around the initial impact of any physical activity media campaign. Levels of recognition and awareness are the initial impact measures used in campaign evaluation. In an international review, Cavill [2] noted that, among 11 physical activity campaigns, a median of 73% of the populations, when prompted, recognized the campaign or main theme. These were almost all short term mass communications, and they measured awareness immediately after the main campaign. By contrast, the original ParticipACTION showed even higher community recall, despite being a set of interrelated campaign activities spread over 30 years. A series of unrelated national polls and surveys over 20 years in Canada showed a median of 85% of adults recalled and were aware of the original ParticipACTION campaign [7]. Current prompted awareness of ParticipACTION is around 76% of Canadian adults [19], rates slightly lower than through the original ParticipACTION era. In particular, rates were markedly lower among young adults who were least likely to be exposed to the original campaign, but were high, at around 90% for adults aged 45-64 years who likely experienced several of the interrelated campaign activities of the original ParticipACTION.

Ongoing effective mass media campaigns require a sustained presence, and regular reinforcement or booster campaigns [8]. The original ParticipACTION campaigns are a rare example of this, and the sustained rates of recall are impressive. This remarkable level of sustained community awareness has only been equaled in the past decade in New Zealand, where serial surveys identify >80% community awareness of the integrated national 'Push Play' campaign organized through the Sport and Recreation national agency [Personal communication, SPARC, NZ]. By contrast, the UK now demonstrates what happens if efforts to promote physical activity are not sustained: new data show that less than one in ten of the population in England correctly identified the recommended level and type of physical activity, 8 years after the last national physical activity campaign was stopped [20]. Yet in the UK, government reports continue to emphasize the importance of sustained communication, using the original ParticipACTION as an example [21]. England's new national physical activity strategy even contains a detailed case study quoting the original 60 year old Swede, and encourages the use of similar international comparisons to galvanise the population into action [22].

In summary, this series of papers outline the successful history of the original ParticipACTION, and the immense challenges for the new ParticipACTION. The tasks of re-inventing a mass media communications campaign with an established brand, and to develop a strategic organizing focus for agencies, groups, and institutions promoting physical activity in Canada is a daunting prospect. The need for leadership, for a national physical activity plan, and for sustainable resources is well recognized by stakeholders [11]. The new ParticipACTION, with its strategic foci of communications and collaborative partnerships, as well as sustained revenue generation, may play a major role in this endeavor.

Setting up an integrated evaluation framework, which includes quantitative population surveys and qualitative research [this issue] about the original ParticipACTION and the current landscape of stakeholders and their perspectives, establishes initial benchmarks. These baselines can be compared to the progress and future influence of the new ParticipACTION. Such comparisons may help with future examinations of the impact of the large natural experiment that is the new ParticipACTION. If the new ParticipACTION can achieve even part of its strategic goals, and attain the population reach of its predecessor, then the resultant physical activity participation levels may be expected to improve in Canada for the coming decade.
